# Exploring the Relationship between Brain Plasticity, Migratory Lifestyle, and Social Structure in Birds

**DOI:** 10.3389/fnins.2017.00139

**Published:** 2017-03-27

**Authors:** Shay Barkan, Yoram Yom-Tov, Anat Barnea

**Affiliations:** ^1^Department of Zoology, Tel-Aviv UniversityTel-Aviv, Israel; ^2^Department of Natural and Life Sciences, The Open University of IsraelRa'anana, Israel

**Keywords:** bird migration, brain plasticity, hippocampus, hyperpallium apicale, new neuronal recruitment, nidopallium caudolaterale

## Abstract

Studies in Passerines have found that migrating species recruit more new neurons into brain regions that process spatial information, compared with resident species. This was explained by the greater exposure of migrants to spatial information, indicating that this phenomenon enables enhanced navigational abilities. The aim of the current study was to test this hypothesis in another order—the Columbiformes – using two closely-related dove species—the migrant turtle-dove (*Streptopelia turtur*) and the resident laughing dove (*S. senegalensis*), during spring, summer, and autumn. Wild birds were caught, treated with BrdU, and sacrificed 5 weeks later. New neurons were recorded in the hyperpallium apicale, hippocampus and nidopallium caudolaterale regions. We found that in doves, unlike passerines, neuronal recruitment was lower in brains of the migratory species compared with the resident one. This might be due to the high sociality of doves, which forage and migrate in flocks, and therefore can rely on communal spatial knowledge that might enable a reduction in individual navigation efforts. This, in turn, might enable reduced levels of neuronal recruitment. Additionally, we found that unlike in passerines, seasonality does not affect neuronal recruitment in doves. This might be due to their non-territorial and explorative behavior, which exposes them to substantial spatial information all year round. Finally, we discuss the differences in neuronal recruitment between Columbiformes and Passeriformes and their possible evolutionary explanations. Our study emphasizes the need to further investigate this phenomenon in other avian orders and in additional species.

## Introduction

The phenomenon of new neuronal recruitment in the adult avian brain has been extensively studied during the last few decades. Many of these studies have indicated a relationship between the amount of new neuronal recruitment and the amount of new information that the brain has to acquire, for example during song learning and production, social interactions, and food hoarding (reviewed in Barnea and Pravosudov, 2011). However, so far, only a few studies have investigated whether a relationship exists between new neuronal recruitment and migratory behavior as the latter exposes migrating birds to more spatial changes than resident ones. For example, LaDage et al. (2011) showed that migratory adult white-crowned sparrows (*Zonotrichia leucophrys gambelii*) recruit more new hippocampal neurons than their residential subspecies (*Z. l. nuttalli*). More recently, we extended this examination (Barkan et al., 2014), looking at two brain regions known to be involved in processing spatial information—the hippocampus (HC) and the nidopallium caudolaterale (NCL), and compared new neuronal recruitment in these regions between two closely-related species—the migratory reed warbler (*Acrocephalus scirpaceus*) and the residential clamorous warbler (*A. stentoreus*). Similarly to LaDage et al. (2011), our study, which was carried out under semi-natural conditions, found more new neurons in both the HC and NCL of the migrant species, compared to the resident one. We suggested that this phenomenon enables the enhanced navigational abilities that are required for the migratory lifestyle.

Most of the studies on new neuronal recruitment in birds have focused on passerine species. Hence, in the current study we aimed to extend the investigation via another avian order—the Columbiformes—and more specifically, in relation to migratory behavior, by comparing migrant and resident species. In this avian order, many studies have used homing pigeons (*Columba livia domestica*), a species with supreme navigational abilities (Papi et al., 1989; Wallraff, 1996, 2001; Odetti et al., 2003; Bingman et al., 2004, 2005; Biro et al., 2007; Jorge et al., 2010; Mehlhorn et al., 2010). These studies indicated that homing pigeons rely on several external cues for orientation and navigation, such as visual landmarks, sun compass, magnetic fields, olfactory cues and more. Motivation and experience were also found to be important for successful homing (reviewed by Mehlhorn and Rehkamper, 2009). In this respect, experience must include learning and memory mechanisms in order to improve navigation abilities and maintain cognitive maps (inherited or acquired by experience). These mechanisms have been suggested as a tool for spatial orientation of long-distance migration in birds (Wiltschko and Wiltschko, 1999). However, to the best of our knowledge, no study has yet examined whether a relationship exist between migratory behavior and new neuronal recruitment in brains of non-passerine species. The only study that recorded neuronal recruitment in Columbiformes used ring doves (*Streptoplia risoria*), and found an overall age-related decline of neuronal recruitment in the forebrain, and primarily in the nidopallium caudal and hyperstriatum regions (Ling et al., 1997).

Accordingly, we aimed to extend our previous study (Barkan et al., 2014) and investigate whether the positive correlation that we had found in passerines, between migratory lifestyle and neuronal recruitment, exists also in non-passerine species. If so, we hypothesized that, as in passerines, new neuronal recruitment into relevant brain regions will be more pronounced in migrant species that travel long-distances twice a year, than in resident ones. In addition, we sought to determine whether differences in neuronal recruitment, if found, would follow a seasonal pattern that will correlate with the migration periods. More specifically, we predicted that neuronal recruitment will increase during or prior to the migrating seasons (spring and autumn), in comparison to summer, the non-migrating season. However, we are aware of possible alternative hypotheses, because others have found, for example in bats, no indication of a correlation between neurogenesis and phylogenetic relationships, foraging behavior, habitat, or diet (Amrein et al., 2007). Such studies argue that the widely accepted notion that neurogenesis has an important role in spatial abilities has to be considered carefully (Amrein and Lipp, 2009).

In order to test our hypothesis, we focused our attention on three brain regions: the HC, NCL, and hyperpallium apicale (HA). The roles of the HC and NCL in processing spatial information were described in our previous study (Barkan et al., 2014; and see also the recent review by Herold et al., 2015). The third brain region, HA, is part of the Wulst, a brain region considered to be homologous to the mammalian visual cortex, although some of its structures evolved independently in birds and mammals (Medina and Reiner, 2000). The Wulst has been suggested to be involved in sun-compass-guided behavior (Budzynski et al., 2002) and in learning or acquisition of familiar landmark navigation (reviewed by Bingman et al., 1998) in homing pigeons. Another function that had been suggested for the HA, together with cluster N neurons, is in assisting in orientated magnetic-compass-guided behavior in nocturnal migrants (Mouritsen et al., 2005). The assumption is that through this pathway birds are able to perceive the geomagnetic field and follow it to navigate in the dark (Heyers et al., 2007; Zapka et al., 2009).

We used two species from the Columbiformes order: the laughing dove (*Streptopelia senegalensis*) and the turtle dove (*S. turtur*). These species were chosen because they are closely related and similar in their behavioral ecology, but the former is resident in Israel while the latter is a migratory species. Their similar behavioral ecology is expressed in both species occupying a wide range of Mediterranean habitats, including agricultural areas and well-vegetated plains. Both species are granivorous, but laughing doves tend to inhabit urban areas and their outskirts, whereas turtle doves prefer semi-open landscapes with trees, orchards or citrus groves (Shirihai et al., 1996). In Israel, laughing doves breed from February to July and their nesting sites vary greatly, from tree-tops to house windowsills, with a minimum of 100 m between nests. Turtle doves breed from April to mid-September and, unlike laughing doves, tend to nest in close proximity to each other, only a few meters apart. Breeding populations arrive in Israel from Africa during spring (mid-March to mid-June) and remain until the end of autumn (mid-September to mid-October), when they gather in large pre-migration flocks and then depart for their winter quarters (Shirihai et al., 1996). Turtle doves winter mainly in northern and central Africa, although on rare occasions they have been recorded as far south as Botswana and South Africa (Cramp and Perrins, 1994; Lepage, 2014; Barkan et al., 2016). In both species we recorded, in spring, summer, and autumn, the number of new neurons in the three brain regions that are thought to be relevant for the processing of spatial information—the HA, HC, and NCL. We hypothesized that, similar to our previous findings in passerines (Barkan et al., 2014), new neuronal recruitment in these brain regions would be higher in the migrant turtle dove than in the resident laughing dove, and that this pattern will correlate with the migration seasons.

## Materials and methods

### Experimental design

Turtle doves and laughing doves were collected under the Israel Nature and National Parks Protection Authority permit (2005/24706). The study was approved by the Tel-Aviv University Institutional Animal Care and Use Committee (permit L-06-008) and was carried out in accordance with its regulations and guidelines regarding the care and use of animals for experimental procedures. Birds were caught at three times during the year: February to mid-April (spring), June to mid-July (summer), September to mid-November (autumn), and Table 1 presents the sample sizes for each species, season, sex and age. Turtle doves were caught in the Beit She'an valley (35°31′E; 32°24′N) using mist nets, and laughing doves were caught by using 1^*^1^*^1 m trap cages built with one directional corridor entrance, and lured with food bait in the Zoological Gardens at Tel-Aviv University (34°48′E; 32°6′N). Upon capture, birds were banded with plastic colored rings (A.C. Hughes LTD., UK) and weighed to the nearest 0.5 g using a 300 g spring scale (Pesola AG, Switzerland). In addition, birds were aged according to plumage and iris color (Shirihai et al., 1996; personal knowledge), and transferred to outdoor aviaries in the Botanical Gardens of Tel-Aviv University. Each bird was held individually and injected, on three consecutive days, into the pectoral muscle with 10 μl/g body mass of 5-bromo-2-deoxyuridine (BrdU; SigmaUltra, Sigma, diluted 10 mg/ml in sterile water). BrdU is a cellular birth-date marker and our doses were similar to those previously used to study adult neurogenesis in birds (Wang et al., 2002; Pytte et al., 2007; Adar et al., 2008). Birds were kept in their aviaries for 5 weeks, to allow enough time for neurons born at the time of treatment to migrate to their final destination and undergo final anatomical differentiation (Alvarez-Buylla et al., 1988; Kirn et al., 1999). Aviaries were 3^*^2.5^*^1 m in size and spaced 1 m apart. The walls of the aviaries were covered halfway up with burlap and the roof was of opaque plastic, to allow visual and auditory contact between individuals, while at the same time providing some isolation, to reduce possible stress. Birds were exposed to natural illumination (10.1–14.7 h of light/day) and temperature (ranging between 12 and 30°C seasonally). Food and water were provided *ad libitum* and consisted of sorghum, milled corn, wheat seeds, crushed eggs, and mineral stones (Redstone,Versele-Laga, Belgium). As an indirect measure of the general health of the birds, we weighed each individual upon capture, then 3 days and 3 weeks later, and on the day of perfusion.

**Table 1:** **Sample sizes, divided by species, seasons, and ages**.

**Species**	**Season**	**Adults**		**Juveniles**		**Total**
		**Males**	**Females**	**Males**	**Females**	
Laughing dove	Summer	1	3	1	4	9
(resident)	Autumn	4	2	3	2	11
	Spring	5	4	1		10
Turtle dove	Summer	6	2		2	10
(migrant)	Autumn	1				1
	Spring	6	2	2		10

### Histology and immunohistochemistry

Birds were killed with an overdose of anesthesia (0.06 ml of ketalar diluted 10 times followed by 0.06 ml of xylazine per gr/body mass) and fixed with an intracardiac perfusion with saline (0.9% NaCl) followed by 4% paraformaldehyde in 0.1 M phosphate buffer (PB; pH 7.4). Sex was determined by gonadal inspection, brains were removed, weighed, transferred into PB in 4°C, and then within 1–4 days they were dehydrated in alcohols, embedded in polyethylene glycol, blocked and cut transversely to 6 μm thick sections. Every 20^th^ section (intervals of 120 μm between sections) was mounted on slides (Superfrost/PLUS), using a solution of 0.1% BSA (albumin bovine, minimum 98%) in PBS. Sections were incubated for 10 min in 0.01 M citrate buffer (pH 5.6–6) at 90–95°C to denature the DNA (to make BrdU more accessible to the antibody), washed in PB for 5 min, and incubated for 2 min in weak pepsin (pepsin stock; Sigma, 2.5% in PBS 1M) diluted 1:19 in 0.1N HCl at room temperature (RT). After washing (PB, 3 times, 5 min each), sections were incubated in 3% H_2_O_2_ in PB for 20 min and then washed with PB twice, 5 min each. Blocking of non-specific binding sites was done by incubating the sections for 1 h in blocking buffer (PB containing 10% normal horse serum and 0.3% Triton X-100). Then, sections were incubated for 48 h at 4°C, with primary antibodies: rat anti-BrdU IgG2a (Serotec; diluted 1:200) and neuronal specific antibody, anti-HuC/HuD mouse IgG2b, monoclonal 16A11 (Invitrogen; diluted 1:200). Then sections were washed and incubated for 2 h at RT with the secondary antibodies: F(ab)2 donkey anti-rat IgG-Cy3 (Jackson ImmunoResearch; diluted 1:200), which stains BrdU cells with fluorescent red and Alexa Flour 488 donkey anti mouse IgG (H+L; Invitrogen; diluted 1:200) which stains neurons with fluorescent green. Then, sections were washed 3 times for 5 min each with PB, coverslipped with Aqua-Poly/Mount glue (Polysciences), and kept in the dark at 4°C until mapping.

### Brain regions, mapping, and quantification

**Figure 1:**
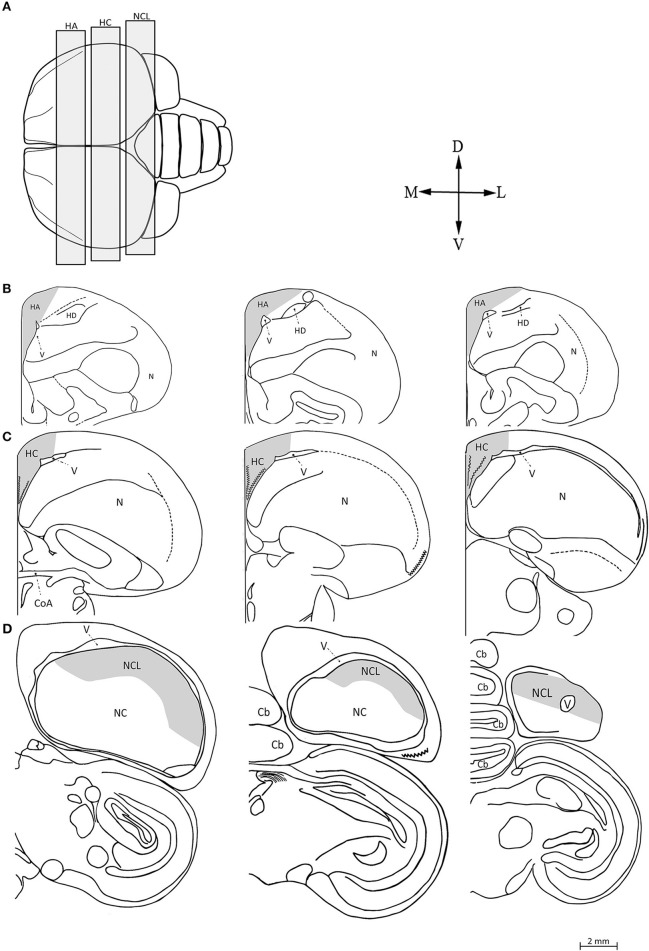
**Schematic views of the three investigated brain regions. (A)** Top view of the brain: rostral is to the left, caudal is to the right. We indicate the range within which frontal sections were taken from the hyperpallium apicale (HA), hippocampal complex (HC), and nidopallium caudolaterale (NCL). Seven sections were sampled along the rostro-caudal axis of each brain region (for details, see text), three of which are shown here: the most rostral, the middle, and the most caudal (from left to right), in HA **(B)**, HC **(C)**, and NCL **(D)**. Abbreviations: Cerebellum (Cb), Commissura anterior (CoA), Hyperpallium Dorsal (HD), Nidopallium (N), Nidopallium caudale (NC), Lateral ventricle (V). Orientations: Dorsal (D), Lateral (L), Ventral (V), and Medial (M). Adapted from Karten and Hodos (1967).

We focused our attention on three brain regions: the hyperpallium apicale (HA), hippocampal complex (HC), and nidopallium caudolaterale (NCL) (Figure 1A). The borders of the brain regions and anatomical positions noted below were defined according to the atlas of the pigeon brain (Karten and Hodos, 1967). We do not know whether pigeons (on which the landmarks were based) and our investigated dove species have proportionally identical brain regions on the rostral-caudal plane. Therefore, in our mapping we wanted to ensure that: (1) our sampling does not extend outside the borders of the region that we mapped; and (2) we avoid overlapping or inclusion of part of another brain region that we sampled. To achieve these goals, we used a conservative approach, so that all the sections that we mapped, in each brain region, were well within it, and did not reach its outmost caudal or rostral extents as identified in the atlas of the pigeon brain. This method resulted in the fact that we did not map the whole region rostral-caudally, but on the other hand it ensured that we mapped only sections that are within this region, and also that these sections do not include parts of another brain region that we sampled. For more specific descriptions, see below.

Accordingly, within each region we defined the rostal and caudal sections that we sampled, and between them we sampled additional ones. Ideally, one would want to sample as many sections as possible, but given the number of birds involved and time limitations, we conducted preliminary mapping on one brain, to determine how many sections we need to map in order to representative results. Therefore, in this brain we mapped about 10 sections per region, and then compared the results that were obtained from these 10 sections to results that we got by including, each time, one less section in our calculations (i.e., by including nine sections, then eight sections, and so on). Based on this preliminary mapping test we settled for total of seven sections per region, which is similar and sometimes even more than the number of sections that we used in previous studies that investigated the same brain regions (e.g., Adar et al., 2008; Barkan et al., 2014). These sections were at fixed and constant anatomical locations across the rostro-caudal axis, so that in all brains, each of the seven sampled sections were exactly in the same rostral-caudal position (with an overall average interval of 240 μm between each pair of sections).

#### Hyperpallium apicale (HA)

**Figure 2:**
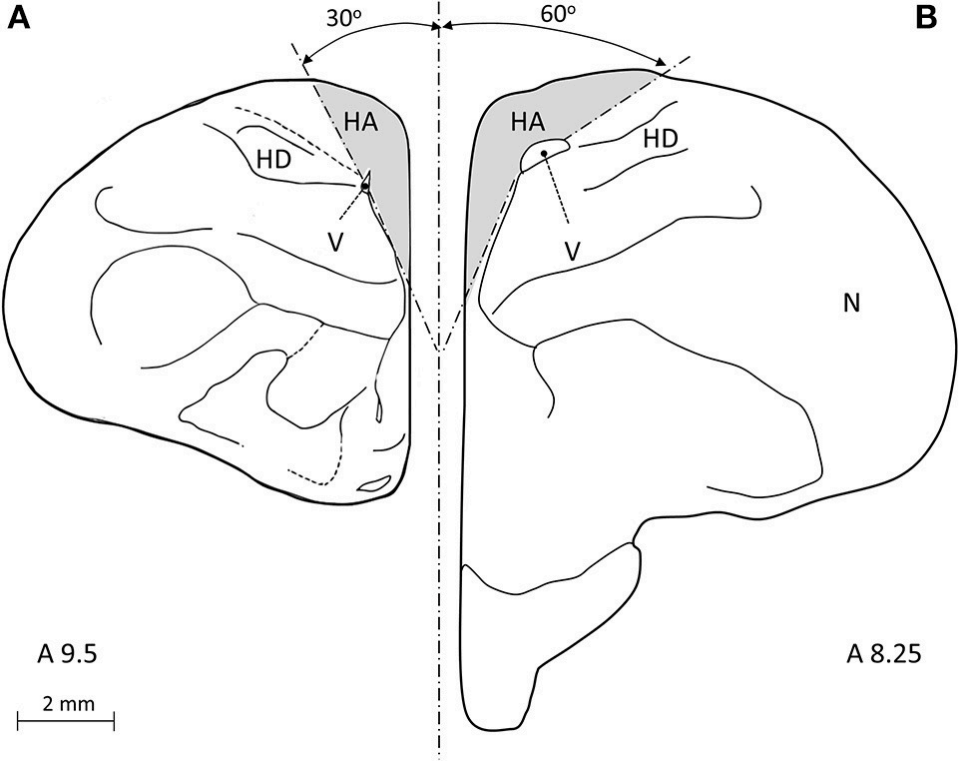
**A frontal view of a pigeon brain (adapted from Karten and Hodos, 1967), is used to illustrate our definition of the lateral boundary of the hyperpallium apicale (HA). (A)** In rostral sections (#1–4) we drew an arbitrary line from the dorsal tip of the lateral ventricle (V) to the dorsal surface of the brain at 30° relative to the midline. **(B)** In caudal sections (#5–7) we applied the same method but at 60° relative to the midline.

The atlas of the pigeon brain defines the rostral-caudal extents of the HA as A14.50 and A7.50, respectively. Therefore, following our conservative approach that is described above, we restricted our sampling so that it was well within the HA, as follows: The most rostral section corresponded to level A9.5 in the atlas of the pigeon brain and the most caudal section was defined 500 μm rostrally to the CoA, corresponding to level A8.25 in the same atlas (Figure 1B). By doing so we ensured that we sampled well within the HA, and in addition do not include parts of the HC (which, according to the atlas, its most rostral extent is A8.00, while we started sampling the HA even more rostrally than that). In each section, the ventral boundary was defined by the wall of the lateral ventricle, the medial boundary by the midline, and the dorsal one by the surface of the brain. The lateral boundary was indistinguishable by our staining methods and was therefore defined arbitrarily, as follows: in the rostral sections (#1–4) we drew a line between the dorsal tip of the lateral ventricle and the dorsal surface of the brain at 30° relative to the midline (Figure 2A). The same method was applied in the caudal sections (#5–7), but at 60° relative to the midline (Figure 2B).

#### Hippocampal complex (HC)

The atlas of the pigeon brain defines the rostral-caudal extents of the HC as A8.00 and A3.25, respectively. According to the same conservative approach that we mentioned above, our sampling of HC was restricted as follows: The most rostral section was defined by the presence of the commissura anterior (CoA), and corresponded to level A7.75 in the atlas of the pigeon brain (Figure 1C). The most caudal section of the HC corresponded to level A6.25 in that atlas. The ventral, dorsal, and medial boundaries in each section were defined according to previously defined criteria (Barnea and Nottebohm, 1994; Barnea et al., 2006).

#### Nidopallium caudolaterale (NCL)

The most caudal section corresponded to level A3.0 in the atlas of the pigeon brain. The most rostral section corresponded to level A4.5 in the atlas, and was defined according to the lateral ventricle along the dorsal part of the brain (Figure 1D). The ventral boundary was indistinguishable by our staining methods and was therefore determined according to the dopaminergic innervation recorded in pigeons by Waldmann and Gunturkun (1993). Since brains of laughing doves and turtle doves differ in size from the pigeon brain, we followed the method described in Barkan et al. (2014) to determine the ventral boundary relative to that in the pigeon brain.

#### Mapping

**Figure 3:**
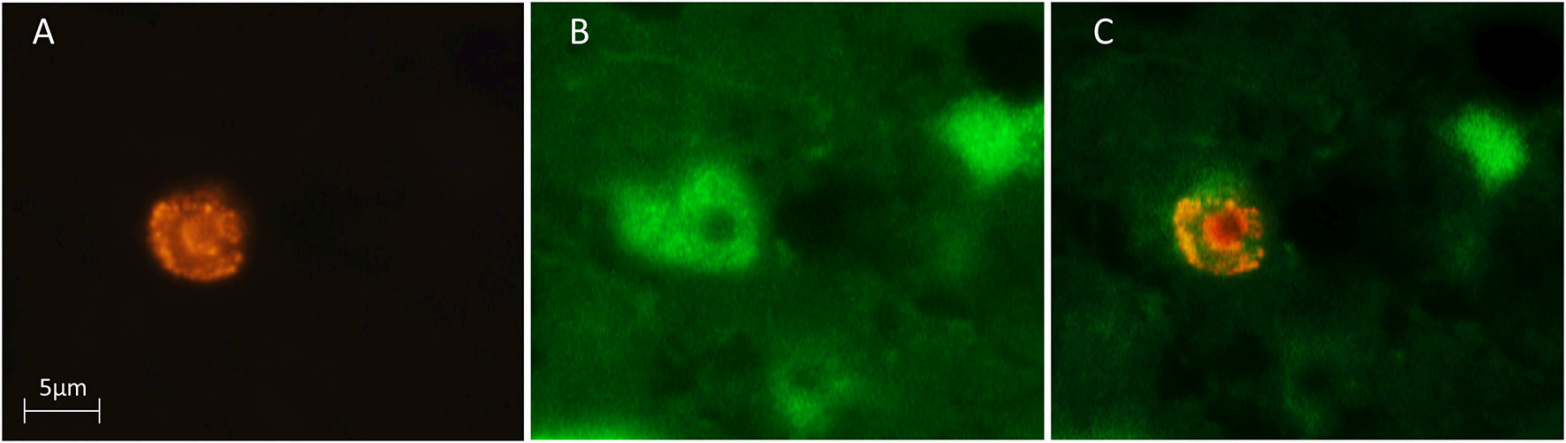
**Three microphotographs of the same field, showing a newly-formed neuron in the hyperpallium apicale (HA) of a turtle dove**. BrdU-labeled cells were identified with a rhodamine filter **(A)** and Hu-labeled neurons were identified with a FITC filter **(B)**. Double-labeled neurons were identified by alternating between these two filters and by using a dual FITC-rhodamine filter to show co-localization of the two markers **(C)**.

**Figure 4:**
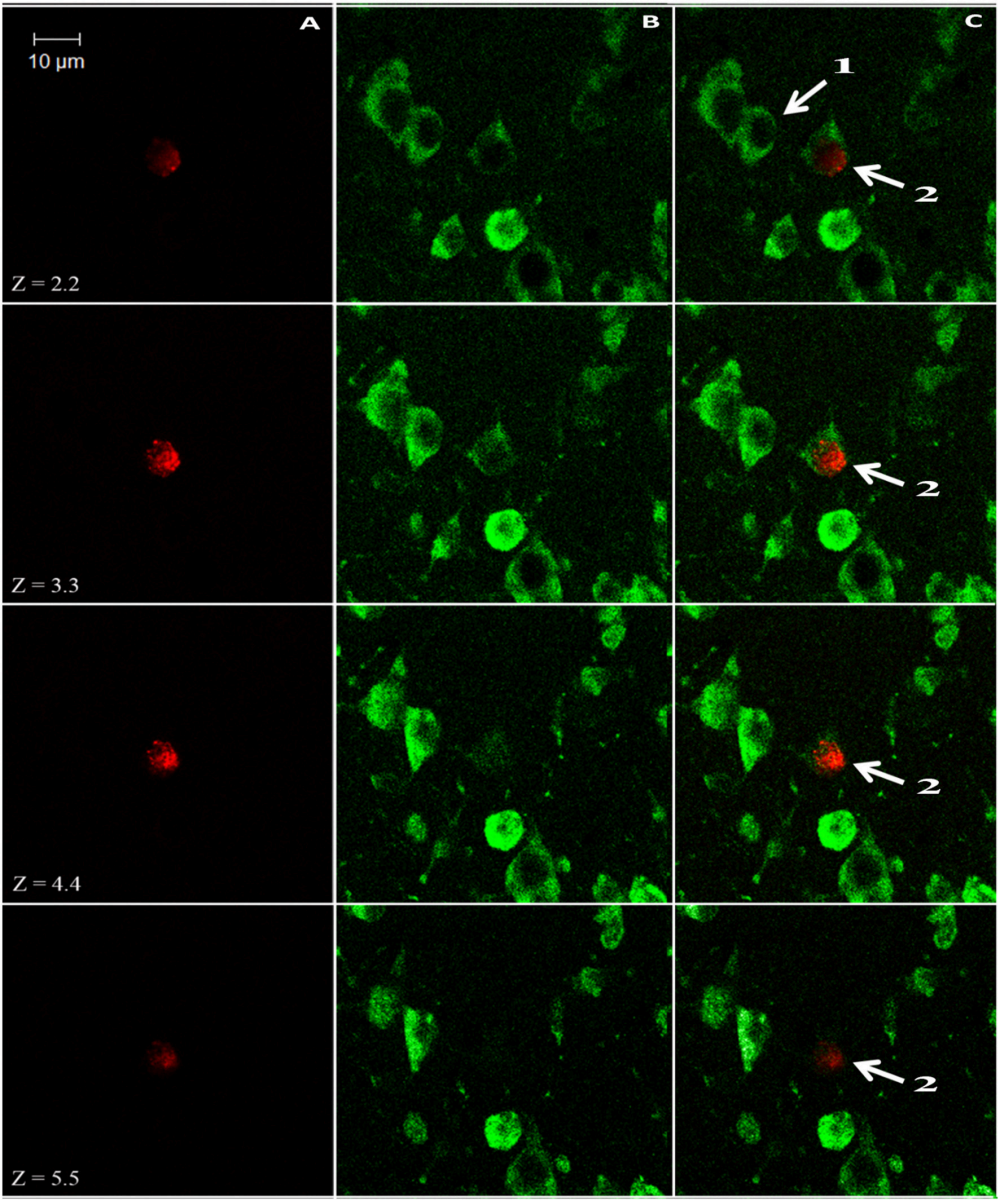
**Z-stack images from the nidopallium caudolaterale (NCL) of a laughing dove (*Streptopelia senegalensis*), under a confocal microscope**. Neurons display only green cytoplasm (labeled with the endogenous marker HU; e.g., cell 1), whereas new neurons also display red nuclei (labeled with the birth-date exogenous marker BrdU; cell 2); these two markers have to co-localize within the same cell along several Z-positions to validate its identification as new neuron. Images were collected at 1.1-lm intervals. **(A)** Red 543-nm wavelength frame. **(B)** Green 488-nm wavelength frame. **(C)** Combined red and green wavelengths.

We used a computerized brain-mapping system (Stereo Investigator; MicroBrightField Inc.) to draw boundaries of the HA, HC, and NCL, in each section sampled, mark the position of each labeled neuron, count neurons and quantify other neuronal parameters, as described below. All sections of all the brains were mapped and analyzed by only one person (SB). Mapping of all the brains was always done blind in relation to season and age. However, due to overall differences in brain sizes between the two species, in some sections the person who did the mapping could identify the species by the size of the section. Ling et al. (1997) found that new neurons are distributed in dove's forebrain without clustering in any particular pattern, and therefore we focused only on the left brain hemisphere. Each of the sampled sections was completely scanned, using a 63X objective and the Stereo-Investigator meander scan probe. Our immunohistochemistry protocol yielded neurons that were stained fluorescent green (with anti-HuC/HuD), and nuclei of new neurons that were stained fluorescent red (with anti-BrdU). Therefore, cells with co-localization of green cytoplasm and a red fluorescent nucleus were identified as new neurons (Figure 3). Accordingly, in each section, we recorded the location of all BrdU^+^ neurons and counted them. To validate co-localization of the two markers, we used a confocal microscope to examine several dozens of such neurons, from the investigated species and from the examined brain regions (Figure 4).

#### Neuronal densities and nuclear diameters of neurons

We also measured nuclear diameters of all BrdU^+^ neurons. From the knowledge of section thickness (6 μm) and mean nuclear diameter of labeled neurons in a certain brain region, using the Abercrombie stereological correction equation (Guillery and Herrup, 1997) we could estimate the number of new neurons per mm^3^ in each section. Data from all mapped sections for each brain region were averaged so that each region was represented by a single estimated number of BrdU^+^ neurons per mm^3^.

In each brain region we also sampled the nuclear diameters of total neurons (BrdU^+^ and unlabeled ones) and estimated their density per mm^3^. This was done in a section that was positioned midway between sections #3 and #4, in order to avoid fluorescence decay in the sections that were used for mapping. In this section, all neurons were counted in 12–18 sampling squares (100 × 100 μm each), randomly chosen by the mapping software, using the fractionator probe. All neurons that were observed within each square or touched two of its boundaries were counted, while neurons that touched the two other boundaries were excluded. In every other sampling square, we also measured the diameters of all neurons. As explained above, these data allowed us to estimate the total number of neurons per mm^3^ in each of the investigated brain regions.

### Statistical analysis

Our data contained four independent fixed variables: migration strategy (migrant, resident); brain region (HA, HC, NCL); season (spring, summer, autumn); age (adult, juvenile). Due to small sample sizes, sex differences were not examined and, therefore, both sexes were pooled. The numbers and percentages of new neurons/mm^3^ that were counted and calculated at seven sections in each brain region were averaged, giving one value for each brain region per bird (depended variables). We could directly compare the number of BrdU^+^ neurons/mm^3^ between species, because densities of total neurons (BrdU^+^ and unlabeled ones) for each brain region were similar between the two species. Accordingly, the results are presented as number of BrdU^+^ neurons/mm^3^. In addition, to allow comparisons with other species in previous studies (ours, as well as from other laboratories), we also calculated and present the percentages of BrdU^+^ neurons out of total neurons/mm^3^.

Effects of the four independent variables on the number and percentage of new neurons/mm^3^ were analyzed by Generalized Linear Mixed Model (GLMM) analysis. This model was chosen because it can deal with gamma-distributed data, as was the case in our study. Total neuronal density and nuclear diameter were tested against the same four independent variables by linear mixed model (MIXED). Model goodness of fit was tested by Akaike information criterion (AIC). Interactions between any of the tested variables are indicated only if they were found to be significant. Significant differences were followed by Bonferroni *post-hoc* comparison, adjusted with multiple comparisons correction. Brain and body mass were log transformed before analysis and tested with one way ANOVA. Tests were performed at 0.05 level of significance (IBM Corp, 2012; SPSS Version 20, Armonk, NY). Results are presented as means (±SE) and sample sizes are provided in brackets.

## Results

### Number of new neurons per mm^3^

**Figure 5:**
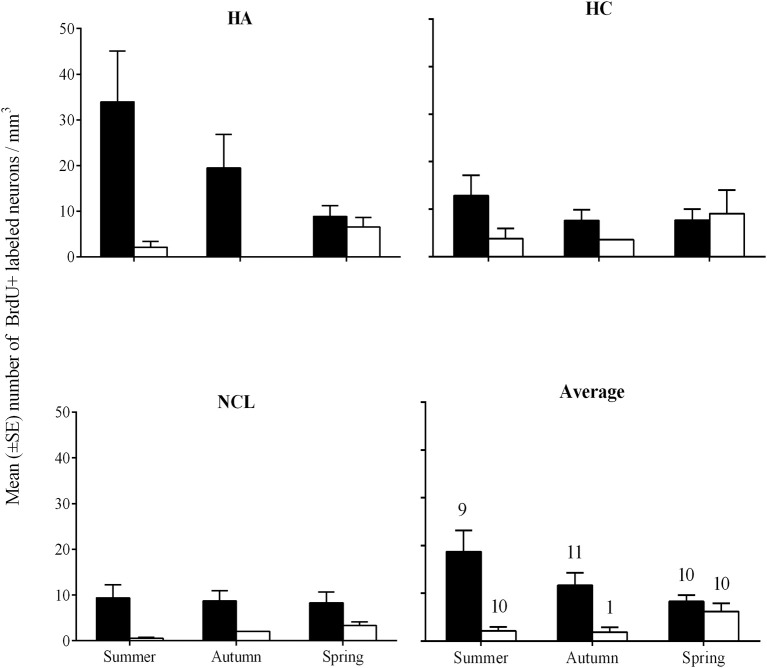
**Mean number (±SE) of new neurons per mm^3^ in the hippocampal complex (HC), hyperpallium apicale (HA), nidopallium caudolaterale (NCL), and the average of the three brain regions (Average), by species and seasons**. Laughing dove: black bars; Turtle dove: empty bars. Sample sizes that are indicated in the Average figure also apply to the three other figures.

Overall we found more new neurons in brains of laughing doves than in those of turtle doves [GLMM, *F*_(1, 144)_ = 11.6, *P* = 0.001]. A correlation between species and season [GLMM, *F*_(2, 144)_ = 3.5, *P* = 0.033] revealed that laughing dove have significantly higher values than turtle dove in autumn and in summer [*post-hoc, P* = 0.07; *P* = 0.017; respectively; Figure 5], while at spring no differences were found between the species. Overall, no significant differences were found between ages, seasons and brain regions. Within each species no differences were found between seasons or brain regions.

### Percentage of new neurons per mm^3^

**Figure 6:**
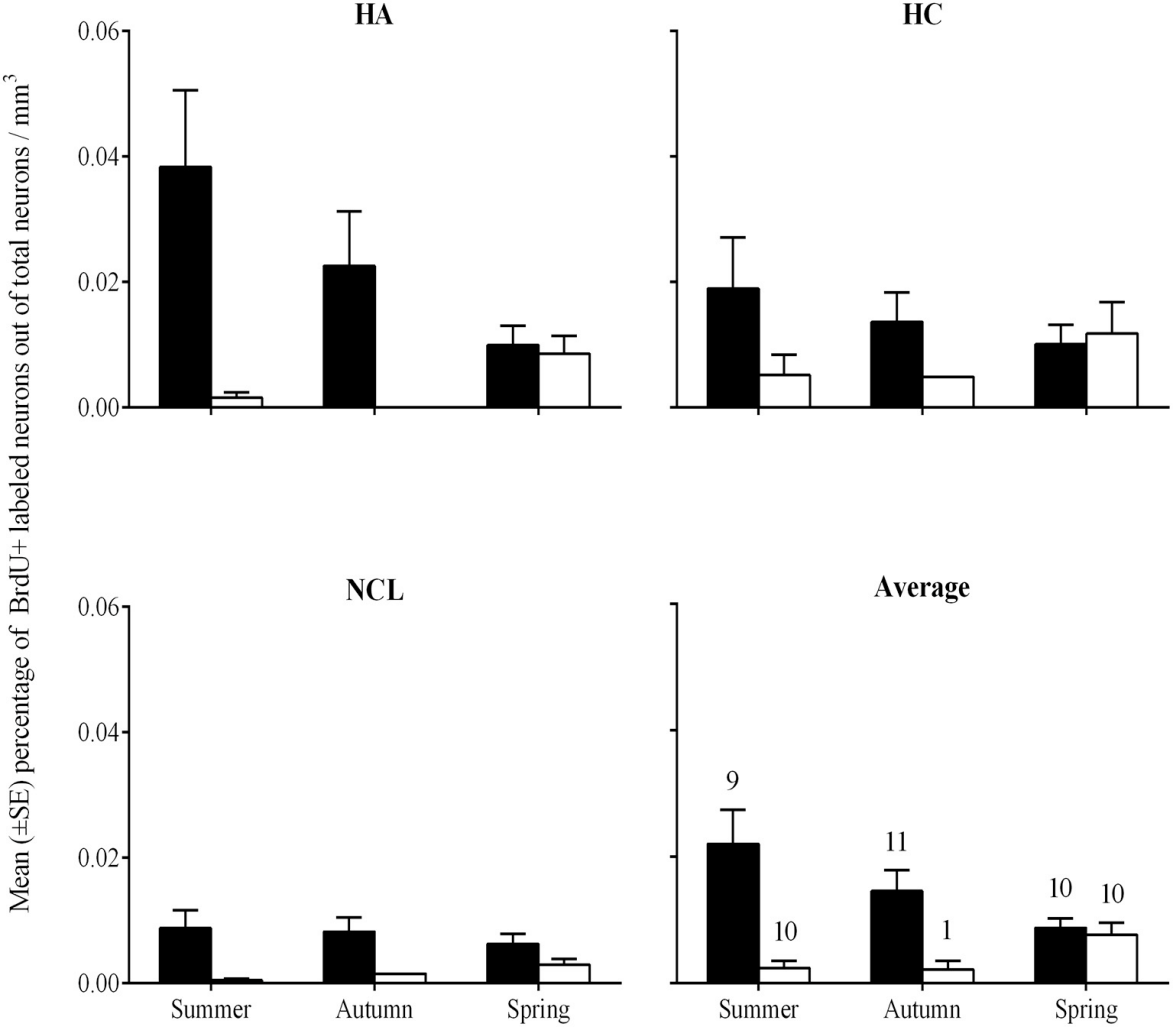
**Mean percentage (±SE) of new neurons per mm^3^ in the hippocampal complex (HC), hyperpallium apicale (HA), nidopallium caudolaterale (NCL), and the average of the three brain regions (Average), by species and seasons**. Laughing dove: black bars; Turtle dove: empty bars. Sample sizes that are indicated in the Average figure also apply to the three other figures.

We found higher percentages of new neurons in brains of laughing doves than in those of turtle doves [GLMM, *F*_(1, 144)_ = 23.3, *P* < 0.001]. A correlation between species and season [GLMM, *F*_(2, 144)_ = 9.64, *P* < 0.001] revealed that laughing dove have higher neuronal recruitment than turtle dove at autumn and summer while at spring no differences were found between the two species (*post-hoc, P* = 0.003; *P* = 0.005; respectively; Figure 6). Overall, significant differences were also found between ages, in both species, with higher values in young individuals than in older ones [GLMM, *F*_(1, 144)_ = 5.1; *P* = 0.026; 0.018 ± 0.001; 0.007 ± 0.001; respectively]. However, due to sample size limitations, we could not test correlations between age and the other variables (seasons, brain regions). No difference was found between the brain regions. Within each species no differences were found between seasons or brain regions.

### Estimated densities of total (BrdU^+^ and non-labeled) neurons per mm^3^

No differences were found between species, season, or age, and pooled data per region, for each species, are presented in Table 2. Significant differences were found between all three brain regions [MIXED, *F*_(2, 146)_ = 48.2, *P* < 0.001], with highest neuronal density in the NCL, intermediate in the HA and lowest in the HC (for all comparisons *P* < 0.001).

**Table 2:** **Mean (±SE) densities and nuclear diameters of total (new and old) and only new neurons, in brains of laughing doves (residents) and turtle doves (migrants).** Pooled data from all seasons are presented by brain region. In brackets for densities—the number of brains sampled; in brackets for diameters—the number of neurons sampled.

**Species**	**Brain region**	**Density of total neurons per mm^3^**	**Nuclear diameter of total neurons (μm)**	**Nuclear diameter of new neurons (μm)**
Laughing dove	HA	96,364 ± 4,376 (29)	9.4 ± 0.17 (2,365)	9.0 ± 0.43 (231)
	HC	73,061 ± 4,288 (39)	10.7 ± 0.26 (2,225)	8.5 ± 0.57 (71)
	NCL	122,051 ± 5,438 (30)	8.8 ± 0.16 (3,062)	9.2 ± 0.37 (1,116)
Turtle dove	HA	104,127 ± 7,395 (21)	8.9 ± 0.3 (1,864)	9.6 ± 0.75 (27)
	HC	77,153 ± 4,619 (20)	10.6 ± 0.35 (1,657)	8.1 ± 0.73 (20)
	NCL	143,146 ± 8,851 (21)	8.1 ± 0.33 (2,218)	8.1 ± 0.41 (181)

### Estimated nuclear diameters of new and total neurons

Due to the relatively low number of new (only BrdU^+^ labeled) neurons found in a given brain region, no statistical comparisons were conducted regarding their nuclear diameters between species, brain regions, seasons, or ages. Pooled data per region, in each species, are presented in Table 2. Total (BrdU^+^ and non-labeled) neuronal nuclear diameters were larger in juveniles than in adults [*t*-test, *t*_(1, 149)_ = 2.76; *P* = 0.006; 10 ± 0.2 (*N* = 3,893); 9.28 ± 0.15 (*N* = 9,528); respectively]. However, for simplicity, in the following comparisons we pooled both age categories and the results for each species are presented in Table 2. No differences were found between the two species in total neuronal nuclear diameter. Brain region comparison found significant differences, with largest neuronal nuclear diameters in the HC, intermediate ones in the HA and smallest in the NCL [MIXED, *F*_(2, 145)_ = 44.1, *P* < 0.001; *Post-hoc* for all comparisons, *P* < 0.001; 10.7 ± 0.2 (*N* = 3,912); 9.2 ± 0.16 (*N* = 4,229); 8.5 ± 0.16 (*N* = 5,280) respectively].

### Body and brain mass

For both turtle doves and laughing doves, no seasonal differences were found in body mass [128.2 g ± 3.46 (*N* = 20); 115.3 g ± 2.88 (*N* = 19); respectively]. During the 5 weeks of captivity, birds' mean body mass decreased only by 1–4.5% of their initial mass and no difference in percentage of body mass loss was found between the species. Similarly, for both species, no seasonal differences were found in brain mass [1.2 g ± 0.01 (*N* = 20); 1.1 g ± 0.02 (*N* = 19); respectively]. Age comparison of brain mass revealed no differences in laughing doves and was not compared in turtle doves due to sample size limitation.

## Discussion

### Neuronal recruitment and sociality

Our previous study (Barkan et al., 2014), which focused on passerine species, supported our hypothesis of a positive relation between migratory lifestyle and new neuronal recruitment in brain regions that are known to process spatial information. An earlier study by others (LaDage et al., 2011) also supports this notion. However, our present study, which used non-passerines from the Columbiformes order, found an opposite outcome, of overall higher neuronal recruitment in relevant brain regions of the resident laughing dove than in those of the migratory turtle dove.

**Figure 7:**
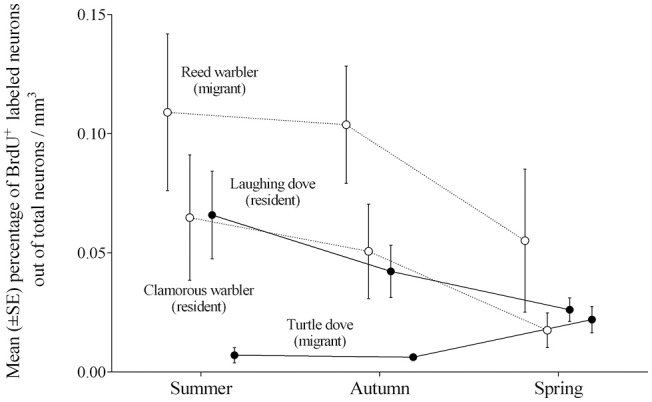
**Mean percentages (±SE) of new neurons per mm^3^ by seasons, combined from the hippocampal complex (HC), hyperpallium apicale (HA), and nidopallium caudolaterale (NCL), in brains of two warblers species (empty circles; the migrant Reed warbler and the resident Clamorous warbler; Barkan et al., 2014), and two dove species (filled circles; the migrant Turtle dove and the resident Laughing dove; current study)**.

The contesting results found between doves and passerines are mainly due to the differences in levels of neuronal recruitment between the migratory species, whereas the resident ones exhibit similar levels, and Figure 7 demonstrates these patterns. We suggest that the differences in neuronal recruitment between the migrating species might be due to their different social structure, with reed warblers being less social than turtle doves. For example, during the breeding season pairs of reed warblers establish separate territories and nest dozens of meters apart from their neighbors (Catchpole, 1972). Similarly, during migration, they move as a wave of individuals, with no apparent organized flocks (Dewolfe et al., 1973; Evans and Heiser, 2004a). Turtle doves, in contrast, are social birds, tending to nest in dense groups only a few meters apart from each other (Shirihai et al., 1996), and migrate in organized flocks (Ash, 1977; personal observation). Hence, could social structure explain the amount of new neuronal recruitment that we found in our studies?

Several studies have focused on the role of sociality in decision-making in migratory birds. For example, homing pigeons form group flights in order to increase their vigilance and reduce predation risk (Gould, 2006). Group flights have also been suggested to improve the birds' navigation as a consequence of the “many wrongs” principle: The pooling of information from many inaccurate compasses yields a single more accurate compass (Simons, 2004). In addition, it had been shown that when conflicts occur between migrating individuals in a group of pigeons, the submissive individuals tend to follow the dominant leaders (Biro et al., 2006; Gould, 2006; Nagy et al., 2010; Flack et al., 2013). Moreover, simulation models of group flights have demonstrated that a few individuals with strong directional preferences can lead large groups of less-well-informed individuals without losing group structure. The model predicted that as group size increases, fewer leaders are needed in order to obtain great accuracy (Couzin et al., 2005). Taken together, these studies suggest that group migration not only improves navigation accuracy compared to solitary migration, but also reduces the navigational efforts invested by each individual in the group. This could, in turn, reduce spatial demands and result in lower levels of neuronal recruitment, as we found in our migrating turtle doves. We are obviously aware that our suggestion that sociality during migration reduces spatial demands has to be tested. This can be done in a future study that will test the spatial memory of these two species.

Another future direction that might be worthwhile taken is in regard to the HC, which is composed of several sub-regions: V-field (that is considered comparable to the dentate gyrus); DM (dorsalmedial); and DL (dorsalateral) (reviewed in Barnea and Pravosudov, 2011, and see also more recent publications, e.g., Abellán et al., 2014; Striedter, 2016). If the counting of new neurons will be adjusted according to these sub-regions, then we might obtain more accurate information regarding levels of neuronal recruitment in the different parts of the HC of migrant vs. non-migrant doves. In addition, it is worth noting that the relationship we suggest, between low neuronal recruitment and high sociality, might be restricted to those brain regions that are known to process spatial orientation tasks. In this study we did not map brain regions which are not involved in spatial tasks. However, in another avian species (zebra finches; *Taeniopygia guttata*), it has been found that in such brain regions (e.g., HVC, Area X, and nidopallium caudale (NC), social complexity increases neuronal recruitment (Lipkind et al., 2002). This was explained by the involvement of these regions in auditory processes and vocal communication, skills that are required in social interactions. Hence, our suggestion is that sociality might have a differential effect on neuronal recruitment in different brain regions, depending on the function of these regions and their contribution to the organism's ability to adjust to different situations.

We realize that other factors might explain the contesting results that we found between doves (this study) and warblers (Barkan et al., 2014), rather than different navigation needs. For example, foraging behavior: granivorous species, such as doves, are not territorial, while most insectivours species, such as warblers, are (Shirihai et al., 1996). Therefore, migrating doves do not need to establish, memorize, and defend territories in order to ensure food supply. Another factor that might explain this opposite outcome between doves and warblers is the fact that doves, which are social and live in groups, can spend less time searching for predators and hence can invest more time foraging, compared with solitary species as the warblers. These differences between doves and warblers exist both in their wintering grounds, as well as in stopover sites en route, and might result in reduced spatial needs in migrating doves, that is manifested by lower neuronal recruitment, compared with migrating warblers. However, because to date only few studies investigated the possible relation between neurogenesis and animal migration, it is needless to say that one has to be cautious when discussing the results. Further research on more species has to be carried out before we can reach reliable conclusions and extract generalizations.

### Neuronal recruitment and seasonality

Several studies revealed a relationship between seasonal behaviors—such as food hoarding (Barnea and Nottebohm, 1994; Patel et al., 1997; Hoshooley and Sherry, 2007; Sherry and Hoshooley, 2010), or song production (Kirn et al., 1991; Alvarez-Buylla and Kirn, 1997)—and seasonal increase in neuronal recruitment. These studies suggested that neuronal recruitment peaks at times of the years when information load peaks. However, testing interactions between behavior and neuronal recruitment is challenging, because it is easy to confound correlation and causation, but difficult to determine the direction of the casual relationships (Brenowitz and Larson, 2015). Do seasonal changes (day length, temperature) cause changes in behavior, which, in turn, cause changes in brain nuclei that are associated with this behavior? Or is the causation opposite (Brenowitz, 2008), so that seasonal environmental changes cause changes in brain nuclei, which, in turn, cause changes in behavior? In relation to singing behavior in seasonal species, Brenowitz (2008) presents evidence for the latter suggestion, arguing that seasonal changes in the song nuclei are predominantly regulated by hormones, and that the subsequent changes in song play a secondary role in reinforcing neuronal changes. Another suggestion is that, in relation to the seasonal food-storing behavior, seasonal changes in the HC may be a consequence of the behavior itself (Herold et al., 2015; Sherry and MacDougall-Shackleton, 2015). Hence, there is still a debate about the factors that control changes in neurogenesis and further examples are needed to draw generalizations.

Our previous work (Barkan et al., 2014) also found seasonal changes in neuronal recruitment in brains of warblers, in regions that are known to process spatial information, where fewer new neurons were recruited in spring compared to other seasons. We interpreted this as being that in spring, when the birds settle in small breeding territories, they require fewer spatial skills, and this might conduce to a reduction in neuronal recruitment, as reflected in a decrease in new neurons, compared to other seasons, when the birds expand their home-range or migrate. However, unlike warblers, in the present study, in both dove species, we found no seasonal changes in neuronal recruitment.

A possible explanation for the different seasonal patterns of neuronal recruitment between doves and warblers could relate to their different territory sizes and the amount of exploration that these species exhibit during the breeding season. Warblers, which are insectivorous (Schluter, 1984), are highly territorial during the breeding season, occupy small territories (average of 332 m^2^ for reed warblers and ~800 m^2^ for clamorous warblers), and spend much time defending them from intruders (Catchpole, 1972; Fazili, 2014). Doves in contrast, are granivorous and rely on patchy food, are less territorial and tend to flock for food searching (Davies, 1976; Schluter, 1984; Shirihai et al., 1996; Temple, 2004). For example, in our study area, doves nested densely in grapefruit orchards and foraged in large flocks in wheat fields, a few hundred meters to several kilometers distant from their nests (Browne and Aebischer, 2003; personal observations). Hence, it can be assumed that doves, which are more explorative than warblers during the breeding season, are exposed to heavier loads of spatial information, and therefore their neuronal recruitment levels in spring, which are similar to those in the other seasons, might serve to facilitate this explorative behavior.

We are aware of the need to be cautious when suggesting that differences in spatial use due to diet and sociality might affect neuronal recruitment. For example, it could be that our captive conditions, although similar for both species, caused them differential stress, because they differ in their natural history and therefore might react differently to captivity. Laughing doves are more urban and more associated with humans, whereas turtle doves prefer open land and normally avoid human presence. Hence, it could be that the latter were more stressed than the laughing doves. Studies in mammals indicated that stress has an inhibiting effect on neurogenesis (reviewed by Mirescu and Gould, 2006). If stress plays a similar role in birds, it could be argued that the reduced neuronal recruitment in brains of turtle doves, compared to laughing doves, is a consequence of captivity. We cannot rule out completely this possibility, because we did not measure levels of stress hormones. This could be tested in a future experiment with a similar set-up, in which glucocorticoid levels will be tested, to assess possible differences in the stress of the two species. Nevertheless, in our current study, the fact that both species lost similar percentages of body mass during captivity might indicate that stress was not a significant concern. In addition, the weight loss was relatively small, much lower than the 10% that is usually accepted as a stress indication, and also much lower than the weight loss of the migrant warblers (average of 9.3%), in which we found higher neuronal recruitment than in the resident ones, although the later lost only 4.7% in average, of their initial body weight (Barkan et al., 2014). Taken together, this evidence indicates that stress might not be a significant concern.

Another factor that should be kept in mind when discussing the different patterns of neuronal recruitment between passerines and non-passerines in relation to migratory behavior, is that Columbiformes and Passeriformes became evolutionally separated over ~85 million years ago (Jetz et al., 2012). Therefore, it could be that over the course of this long period their brain structures and neuronal mechanisms were shaped under different evolutionary pressures rather than migratory or residency lifestyles. Further study is thus needed in order to test our suggested hypothesis.

### Neuronal recruitment and age

Previous studies in birds and mammals have revealed a general decline in neuronal recruitment with age, which is explained as a consequence of the post-natal brain growth and maturation attained in adulthood (reviewed by Barnea and Pravosudov, 2011). This trend was reported in several passerines species (Alvarez-Buylla et al., 1994; Barnea and Nottebohm, 1996; DeWulf and Bottjer, 2002; Pytte et al., 2007; LaDage et al., 2011; Barkan et al., 2014), but in non-passerines there has been only one study (Ling et al., 1997), which reported a similar pattern in the NC of ring doves. According to that study, at the age of 3 months ring doves recruited on average 0.14% of new neurons per mm^2^ in their NC, declining to 0.02% at the age of 6–8 years. We found a similar age effect (between juveniles and adults), in both species, when calculating percentages of new neurons out of the total. However, due to sample size limitations we could not further correlate age with other factors such as seasons or brain regions, comparisons that could add interesting insights upon neuronal recruitment in the aging avian brain.

### Neuronal recruitment in columbiformes and passeriformes

In both bird orders neuronal recruitment occurs in the HA (current study; Hoshooley et al., 2005), HC (current study; Barnea and Nottebohm, 1994), Medial striatum (former named Lobus parolfactorius - LPO; Alvarez-Buylla et al., 1994; Ling et al., 1997), NC (Ling et al., 1997; Barkan et al., 2007) and NCL (current study; Barkan et al., 2014). However, other brain regions exhibit neuronal recruitment in one of the two above orders, but are less developed or absent in the other. For example, HVC, which is an important nucleus in the brain pathway that controls song production and exhibits seasonal new neuronal recruitment in Passerines (Kirn et al., 1994; Rasika et al., 1994; Alvarez-Buylla and Kirn, 1997; Balthazart et al., 2008), is absent in Columbiformes. Similarly, evidence indicates that pigeons use olfactory cues for navigation and have relatively developed olfactory lobes (reviewd by Mehlhorn and Rehkamper, 2009), while most Passerines are assumed to have a poor sense of smell (Cobb, 1968; Evans and Heiser, 2004b). Thus, it would be interesting to compare neuronal recruitment in the olfactory lobes of Passerines and Columbiformes.

### Total neuronal densities

Unlike passerines such as white-crowned sparrows (LaDage et al., 2011), juncos (*Junco hyemalis*; Cristol et al., 2003), or warblers (Barkan et al., 2014), in which migrant birds' brains had higher densities of total neurons than resident birds' brains, no such difference was found in the present study in doves. From this finding, combined with the higher neuronal recruitment found in the brains of laughing doves, we can assume that a faster neuronal turnover occurs in this resident species compared to that in brains of the migrating turtle doves. If this is so, then neurons in the brains of laughing doves survive for a shorter time than neurons in the brains of turtle doves. Barnea et al. (2006) suggested that “if neurons store information which is acquired by experience, then one form of time representation would be how long neurons that encode information will survive, because when these neurons are replaced, any information they might have stored will be probably lost.” According to this notion, the rate of neuronal turnover might regulate memory duration, so that slower turnover results in more prolonged memory in turtle doves and shorter memory in laughing doves. Longer memory duration could be beneficial to migrating birds by enabling them to store orientation information for a longer period. Differences in memory duration were demonstrated for example in migrating garden warblers, which were able to remember feeding sites for up to 12 months, compared to resident Sardinian warblers that remembered such sites for only 2 weeks (Mettke-Hofmann and Gwinner, 2003).

## Overview

In the current study we found that in two dove species, unlike passerines, new neuronal recruitment was lower in the brains of a migrating species compared with a resident one. We suggest that this could be due to the high sociality of these doves, which forage and migrate in flocks, and therefore can rely on the communal spatial knowledge that is acquired by the flock and might reduce individual navigation efforts. This, in turn, might enable reduced levels of neuronal recruitment in brain regions that process spatial information. In addition, we found that, unlike in several passerine species, seasonality does not affect neuronal recruitment in these two dove species. We suggest that the similar amount of neuronal recruitment that we observed in the brains of the doves throughout the year might be due to their non-territorial and explorative behavior, which exposes them to substantial loads of spatial information all year round. We also found an age effect on neuronal recruitment, which is in line with previous studies in other avian species.

Our current findings are novel in shedding light on neuronal recruitment in brains of two species from the Columbiformes, an order that been hardly studied to date in this respect. Moreover, we looked at three brain regions that are known to process spatial information—the HC, HA, and NCL. Consequently, we discuss the differences in neuronal recruitment patterns that we observed between doves (the present study) and warblers (Barkan et al., 2014), and their possible evolutionary explanations. We suggest that the hypothesis, confirmed in previous studies, that neuronal recruitment in relevant brain regions is expected to be higher in migratory species, might not hold true in all cases. Nevertheless, the idea in principle that the need for more spatial information correlates with higher recruitment hold true, but it can depend on various aspects of the general biology of the species under study, such as sociality, amount of territoriality, foraging patterns, and levels of predation risks. Hence, the findings from our study suggest that variability might exist among different avian orders in patterns of brain plasticity, and emphasizes the importance of further investigation of this phenomenon in other avian orders and in more species, for a better understanding of its role in different lifestyles. This is in line with the growing acknowledgment regarding the remarkable cognitive abilities of birds and their usefulness as a comparative model in order to study the translation of nervous system organization into representations of space that can support navigation and memory (see review by Herold et al., 2015).

## Author contributions

AB, YY, and SB conceived this work, SB conducted the experiments, all authors wrote the paper and contributed to ideas.

## Funding

This study was supported by The National Institute for Psychobiology in Israel and The Research Fund of The Open University of Israel.

### Conflict of interest statement

The authors declare that the research was conducted in the absence of any commercial or financial relationships that could be construed as a potential conflict of interest.
